# Editorial: Women in science - hematology 2023

**DOI:** 10.3389/fmed.2024.1402357

**Published:** 2024-04-09

**Authors:** Eleni Gavriilaki, Chien-Ling Huang, Lalitha Nayak

**Affiliations:** ^1^Hematology Department, Bone Marrow Transplantation (BMT) Unit, G. Papanicolaou Hospital, Thessaloniki, Greece; ^2^Department of Health Technology and Informatics, The Hong Kong Polytechnic University, Hong Kong, China; ^3^Department of Medicine, School of Medicine, Case Western Reserve University, Cleveland, OH, United States

**Keywords:** medicine, hematology, STEM, histo-hematology, molecular-hematology, UNESCO

This Research Topic is part of the Women in Science 2023 series. Other titles in the series include Women in Science—Gastroenterology 2023, Women in Science—Regulatory Science 2023, and Women in Science—Rheumatology 2023. Building on the success of Women in Science—Hematology 2021 (1), we are pleased to present a new volume for 2023 of this Research Topic. While the proportion of women and men in science, technology, engineering, and mathematics (STEM) at undergraduate levels is relatively equal, there is a noticeable lack of representation of women in senior positions in public health. According to 2016 data from the UNESCO Institute for Statistics (UIS) data, women represent < 30% of researchers in STEM and < 4% of Nobel Prizes for science. In Hematology, many highly influential and successful women are contributing to the field and tackling important questions. Nevertheless, female scientists are still underrepresented in various aspects of academic life. Several initiatives have been recently created to increase the visibility of women in science, such as awards for women in STEM. However, evidence indicates that gender bias is still present throughout many scientific disciplines.

This Research Topic aims to highlight female contributions to medicine, specifically in the field of Hematology and will therefore welcome:

• General perspectives on a specific field of research inspired, started or sparked by a woman.

• Articles celebrating outstanding female researchers and their contributions to computer science and public health.

• Public Health studies led by women researching technology and health.

All articles considered for this Research Topic were written by female scientists as first or last authors. Early career scientists were encouraged to team up with senior female colleagues After a rigorous peer review process, seven articles were published.

(i) Palomo et al. have reviewed mechanisms and suggested biomarkers of endothelial damage. A better understanding of endothelial injury is necessary for future preventive or therapeutic strategies.

(ii) In the context of paroxysmal nocturnal hemoglobinuria (PNH), Du et al. have reported a rare case of hemorrhagic esophageal varices with portal vein thrombosis that was treated with transjugular intrahepatic portosystemic shunt (TIPS).

(iii) In a collaboration of first and last female authors, Kalamara et al. have performed a meta-analysis of observational studies, highlighting the significant association of splenectomy with thrombosis but not with pulmonary hypertension in patients with transfusion-dependent thalassemia.

(iv) In the life-threatening field of thrombotic microangiopathies, Gavriilaki et al. reported the safety and efficacy of caplacizumab in immune thrombotic thrombocytopenic purpura (TTP), including cases refractory to plasma exchange, re-administration, and cases without previous plasma exchange treatment.

(v) In an interesting review, Kaddoura et al. provided a practical guide to the management of cardiopulmonary toxicities related to tyrosine kinase inhibitors in patients with chronic myeloid leukemia.

(vi) From the nursing perspective, Yang et al. reviewed the management of treatment-related venous thromboembolism in multiple myeloma. Through this retrospective analysis, the researchers identify the necessity for effective risk assessment models to provide a more accurate prediction of thrombosis.

(vii) Xu et al. (2) demonstrate how the addition of two condition-specific bolt-on items can increase performance on the EQ-5D-5L in patients with hemophilia.

(viii) Last but not least, Shafqat et al. summarized the role of neutrophil extracellular traps in diabetes mellitus complications and highlight the importance of clinical trials to translate the results of these studies.

Considering the multi-disciplinary character of this Research Topic, we hope that it will inspire female researchers and clinicians to continue their explorations into novel advances in their fields.

## Author contributions

EG: Writing – review & editing, Writing – original draft. C-LH: Writing – review & editing, Writing – original draft. LN: Writing – review & editing, Writing – original draft.
